# Supplementary figures and images for: Diagnostic accuracy of three commercially available one step RT-PCR assays for the detection of SARS-CoV-2 in resource limited settings
Source: PLoS One. 2022 Jan 20;17(1):e0262178. doi: 10.1371/journal.pone.0262178 (PMC8775315; doi:10.1371/journal.pone.0262178)

### Annex

**Diagram 1-** Flow of Participants who participates in this study, Ethiopia, 2021.


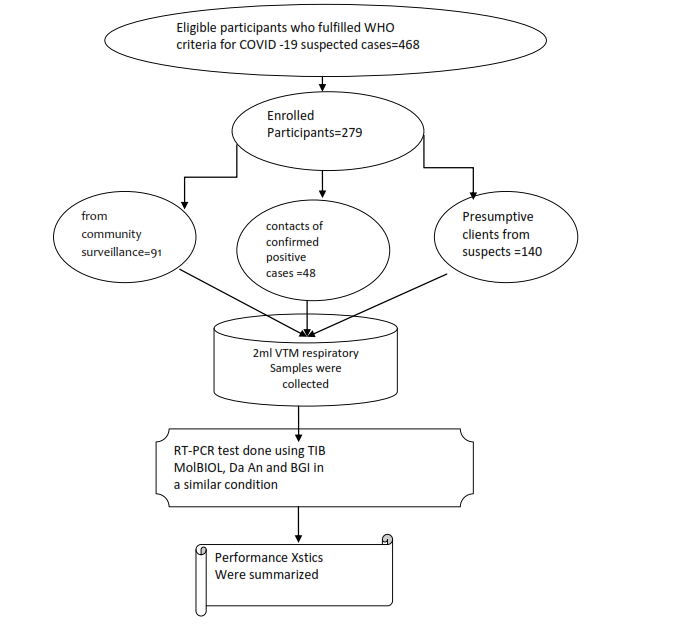

Supplement: S1 Annex — (DOCX) [file pone.0262178.s002.docx]
